# Novel Concepts for Graphene-Based Nanomaterials Synthesis for Phenol Removal from Palm Oil Mill Effluent (POME)

**DOI:** 10.3390/ma16124379

**Published:** 2023-06-14

**Authors:** Kehinde Shola Obayomi, Sie Yon Lau, Michael K. Danquah, Jianhua Zhang, Tung Chiong, Masahiro Takeo, Jaison Jeevanandam

**Affiliations:** 1 Department of Chemical Engineering, Curtin University, CDT 250, Miri 98009, Sarawak, Malaysia; 2 Institute for Sustainable Industries and Liveable Cities, Victoria University, Werribee, VIC 3030, Australia; 3 Department of Chemical Engineering, University of Tennessee, Chattanooga 615 McCallie Ave, Chattanooga, TN 37403, USA; 4 Department of Applied Chemistry, Graduate School of Engineering, University of Hyogo, 2167 Shosha, Himeji 671-2280, Hyogo, Japan; 5 CQM—Centro de Química da Madeira, Universidade da Madeira, Campus da Penteada, 9020-105 Funchal, Portugal

**Keywords:** adsorption, graphene, organic pollutants, phenols, water decontamination

## Abstract

In recent years, the global population has increased significantly, resulting in elevated levels of pollution in waterways. Organic pollutants are a major source of water pollution in various parts of the world, with phenolic compounds being the most common hazardous pollutant. These compounds are released from industrial effluents, such as palm oil milling effluent (POME), and cause several environmental issues. Adsorption is known to be an efficient method for mitigating water contaminants, with the ability to eliminate phenolic contaminants even at low concentrations. Carbon-based materials have been reported to be effective composite adsorbents for phenol removal due to their excellent surface features and impressive sorption capability. However, the development of novel sorbents with higher specific sorption capabilities and faster contaminant removal rates is necessary. Graphene possesses exceptionally attractive chemical, thermal, mechanical, and optical properties, including higher chemical stability, thermal conductivity, current density, optical transmittance, and surface area. The unique features of graphene and its derivatives have gained significant attention in the application of sorbents for water decontamination. Recently, the emergence of graphene-based adsorbents with large surface areas and active surfaces has been proposed as a potential alternative to conventional sorbents. The aim of this article is to discuss novel synthesis approaches for producing graphene-based nanomaterials for the adsorptive uptake of organic pollutants from water, with a special focus on phenols associated with POME. Furthermore, this article explores adsorptive properties, experimental parameters for nanomaterial synthesis, isotherms and kinetic models, mechanisms of nanomaterial formation, and the ability of graphene-based materials as adsorbents of specific contaminants.

## 1. Introduction

The world’s main vegetable oils are derived from palm, soybean, coconut, olive, peanut, sunflower, cottonseed, and rapeseed [1]. Palm oil mill effluent (POME) is a liquid by-product of the crude oil palm production process that contains organic acids as well as phenolic compounds extracted from oil palm fruit and cell wall fragments. POME is an organic acid mixture that also contains hazardous organic matter, expressed in chemical oxygen demand (COD) at concentrations between 45,500 and 65,000 mg/L, which is 100 times more hazardous than residential sewage [2]. Edible oil refining processes generate a large amount of agricultural runoff with effluents that vary in quantity and characteristics between industries and mills [3]. If left untreated, agricultural wastewater would deplete water oxygen levels rapidly due to the presence of organic and nutritional elements, posing a long-term threat to aquatic life and the food chain [1,4]. Improper disposal of industrial waste, particularly palm oil mill effluent (POME), can result in significant environmental problems. According to Chantho et al. (2016), the production of one ton of crude oil generates around 2.5 tons of POME, which contains high concentrations of bio-recalcitrant phenolic compounds [5]. In recent times, the rapid expansion of the edible oil industry, increased public awareness, and tightened environmental legislation have led to the need to treat agricultural effluents before they are discharged into natural water sources [6]. Environmental policies and regulations have been helpful in preventing the accumulation of waste in agricultural systems. However, there is a growing demand to convert agricultural waste into valuable products [7]. Phenolic compounds are organic compounds that are dissociated or non-dissociated and ionize in an aqueous medium with a pH different from water [8]. The presence of phenol in water can pose a serious threat to biological systems due to its toxic and persistent nature. Al-Ghouti et al. (2022) suggest that removing phenolic waste from water and generating a secondary by-product with increased conductivity and salt content is often a difficult task due to the high levels of biomass content. The accumulation of phenol in the food chain can also be a health concern, as it can result in the formation of stable chemical species [9]. At concentrations as low as 1 μg/L in drinking water, phenolic compounds can result in taste and odor problems and be toxic to the function of the human liver and kidney when exposed to higher concentrations for a certain period of time [10].

Several techniques are available for POME treatment; however, not all are suitable as they have varying treatment efficiencies and costs [8]. POME treatment techniques can be classified into physical, chemical, and biological methods [11]. However, these methods have drawbacks such as long assembly and operation periods, high energy usage, expensive maintenance and operation costs, and the generation of sludge, which requires secondary treatment [12]. Finding a low-cost and environmentally sustainable POME waste treatment technique for industrial use is challenging. Therefore, a novel, cost-effective technique for the decontamination of POME from various contaminants, especially phenols, is necessary [13]. The wet extraction technique used in palm oil mills releases contaminants from palm fruit-derived chemicals [14], inhibiting methanogenesis due to certain simple phenolic compounds and polyphenols [2,15]. However, these treatment processes have limitations, such as the requirement of toxic chemicals and a longer decontamination time [16,17]. Adsorption has been proven to be a low-cost, accessible, profitable, and effective method for POME waste effluent treatment compared to other conventional methods. Adsorption is a critical method that emphasizes the interactions between pollutants and adsorbents at the solid-liquid interface [18]. However, limitations such as the toxicity and instability of adsorbents have led to the need for novel adsorbents for pollutant removal applications.

In recent times, nanotechnology has been widely employed in several applications, including wastewater treatment, as nanomaterials have been proven to be highly effective in eliminating inorganic and organic pollutants from wastewater effluents compared to conventional adsorbents due to their low toxicity and small size [19,20,21]. Among nanoparticles, nanosized graphene possesses unique mechanical, physical, and chemical properties that have gained significant attention worldwide for environmental applications [22]. Graphene is used as an adsorbent in wastewater treatment due to its large surface area, improved active sites, large-delocalized electron systems, and superior chemical stability [23]. Recently, nanosized graphene, graphene oxide (GO), and their composite materials have emerged as novel materials beneficial for wastewater treatment applications. GO has been shown to have great adsorptive efficiency due to the abundance of functional groups compared to graphene [24]. GO in its nanoscale form is a two-dimensional material composed of carbon atoms that feature various oxygen-containing functional groups, such as hydroxyl, carboxylic acids, epoxides, or alcohol, which enhance its stability in water (Gallegos-Pérez et al., 2020). The addition of oxygen-containing functional groups, such as hydroxyl, carbonyl, epoxide, phenol, and carboxyl groups, to the graphene oxide structure results in a hydrophilic nature and a modifiable functional group structure (Obayomi et al., 2022). Various composites are synthesized by modifying GO with polymers, metal and metal oxides, or hydroxide nanomaterials, as well as small molecules, to produce GO-composites, which can be utilized as membranes, adsorbents, and catalysts for decontaminating pollutants.

The utilization of nanosized GO as an adsorbent can affect the extent of oxidation via interactions and the presence of hydrogen bonds due to its complex structure [9]. Researchers have shown interest in utilizing GO-based nanocomposites for phenol removal due to their large specific surface areas and various oxygenated functional groups, such as carboxyl and hydroxyl groups, that have a high affinity for the pollutant [25]. Although graphene-based materials are utilized in adsorption studies, the adsorptive capability of GO can be enhanced through surface modification [26]. Hence, graphene-based composites are a great choice for phenolic chemical adsorption. Graphene oxide (GO), a highly oxidative form of graphene with several polar functional groups, has a theoretical surface area that is retained, which allows for its modification and hybridization with other materials [27]. Therefore, graphene-based nanocomposites are identified as an excellent choice for phenolic compound uptake compared to standalone GO. This article provides an overview of conventional synthesis and characterization approaches for GOs and the mechanism of graphene nanomaterial formation, with a special focus on the adsorptive performance of graphene-based nanocomposites as they relate to phenol removal from POME in batch and continuous processes. In addition, the article discusses the phenol removal efficiency of conventional and nanosized GO as well as GO-based nanocomposites and their potential as effective adsorbents in the future. Again, the steps taken by researchers at the pilot scale and laboratory scale were highlighted to achieve an effluent of high-quality water from POME that can be utilized as drinking water.

## 2. Characteristics of Phenol Content in POME

The discharge of toxins from anthropogenic activities into the environment has resulted in one of the major challenges of this century, which is the scarcity of clean water. Phenol is frequently found in industrial wastewater generated by sectors such as oil, gas, medicine, and pesticide production. As it can be harmful, numerous environmental protection agencies have instituted stringent concentration limits for phenol in wastewater to safeguard human health and preserve ecological equilibrium [28]. In ASEAN countries, where palm oil is a significant economic crop, the rapid expansion of the palm oil industry has led to environmental issues caused by the release of large volumes of palm oil mill effluent (POME) into water sources. POME contains high levels of phenolic chemicals, making it a highly contaminated effluent with a colloidal suspension content of 95–96%, total solids of 4–5%, and palm oil of 0.6–7%. The dark brown color of POME is attributed to the oxidation of phenolic components, such as lignin and anaerobically reduced products [29].

Phenol, a white, crystalline, and volatile aromatic chemical compound, is widely found in wastewater from several industries, including oil, gas, medicines, and pesticides, and even at low concentrations, it can affect the taste and odor of water [30]. Phenol is prone to vaporization, resulting in its widespread distribution in the atmosphere, where it can interact with hydroxyl and nitrate radicals and participate in photochemical reactions, generating dihydroxybenzenes and nitrophenol. This process represents the principal mechanism for the removal of phenol from the atmosphere [31]. Factors such as temperature and season, as noted by Møller et al. (2014), can impact the half-life of phenol, which ranges between 2.28 and 22.8 h for reactions with hydroxyl radicals [32]. Phenol is not fully degraded in water due to its short half-life, resulting in its concentration in urban and industrial areas. Moreover, phenols have low sorption due to low partition coefficients between phenol and octanol and minimal water or organic carbon content in soils and sediments, causing them to seep into groundwater close to industrial plants and waste sites [33]. Phenols have rapid biodegradability as they are used as substrates by both aerobic and anaerobic bacteria, with half-life values ranging from 2.7 to 552 h in soils and sediments, depending on their inherent properties [32]. Additionally, phenols can undergo adsorption, volatilization, and oxidation in addition to biodegradation [33]. While phenol is more persistent in water than in air, soil, or sediment, it still degrades in low concentrations. Phenols undergo volatilization, photodegradation, and biodegradation in aquatic environments, with the latter being the most common elimination method. Phenol reacts with hydroxyl and peroxyl radicals in surface waters, with mean half-lives of 100 and 19 h, respectively [33]. However, volatilization often takes more than three months.

Numerous studies have shown that phenol is becoming a concern due to its persistence in the environment [33]. An unintentional spill that occurred in Wisconsin (USA, 1974) is an example of the risk associated with phenol. A concentrated phenol-containing solution contaminated an aquifer for nineteen months, which negatively impacted the health of the people in the affected area. In addition, the high phenol content hindered the biodegradation process of the water [34]. Even though phenol has a short half-life, its continuous release into the environment can have significant impacts on humans and other living organisms. Therefore, it is classified as a priority pollutant by both the US Environmental Protection Agency (USEPA) and the Canadian National Pollutant Release Inventory (NPRI) because it can result in both immediate and lasting effects, even at reduced levels [35]. Human exposure to phenol can occur via inhalation, cutaneous contact, or ingestion of products that are contaminated and can spread rapidly throughout the body once ingested. Phenol can penetrate the membranes of cells and be metabolized into intermediates (e.g., quinone groups) that can interact with protein functions and cause cell death [28]. Symptoms of acute phenol exposure include skin, eye, and mucous membrane irritation, but they can progress to more severe conditions, such as breathing difficulties, weakness in the muscles, lowered body temperature, convulsions, and death, depending on the level of exposure [36]. Abnormal development and behavior in offspring, fertility decline, and fetal body weight reduction have been recorded in animals exposed to phenol [37]. Kulkarni and Kaware (2013) indicate that the concentrations of phenol that can cause negative effects in humans and aquatic species are 10–24 and 9–25 mg/L, respectively [38]. Various conventional strategies, including distillation, chlorination, and absorptive extraction approaches, have been used to eliminate phenol from aqueous media. However, these techniques have certain disadvantages, such as limited efficiency, the ability to generate sludge and harmful byproducts, a high cost, and high energy requirements [39].

Adsorption and advanced oxidative processes (AOPs) have been demonstrated as viable, practical, and environmentally friendly alternatives for removing phenol from water among the advanced remediation technologies. In recent years, tertiary treatments have been successfully used to increase phenol absorption and meet stringent water quality requirements. Advanced oxidation technologies and adsorption are reported as highly viable approaches among these tertiary methods. Adsorption is an advantageous technique for phenol removal due to its simplicity in construction and use, cost-effectiveness, and ability to handle low-concentration fluids [40]. In addition, AOPs have become increasingly popular due to their low environmental impact, fast reaction times, ability to mineralize organic molecules, and complete elimination of pollutants [41]. Combining these approaches is considered a promising option for phenol removal [42]. To date, several chemical-based materials have been utilized as conventional adsorbents for phenol removal applications, including metal oxides [22], activated carbon [43], silica-based aerogels [44], and natural materials such as activated carbon from lignocellulosic agricultural wastes [45], zeolites [46], and rice husk [47]. However, these macro-materials cannot completely remove phenols from environmental conditions with high efficiency due to their larger size. Thus, novel structures or nanosized materials are required to improve phenol removal efficiency. Among these novel structures, graphene oxides (GOs) have recently been reported to possess enhanced ability to remove phenols from contaminated sites due to their unique two-dimensional (2D) morphology [48].

## 3. Synthesis and Characterization of GOs

Graphene is a material that has been widely studied over the last few decades and has been dubbed the “material of the future” due to its exceptional properties. Graphene has a unique honeycomb lattice structure in a two-dimensional plane, where each carbon atom is bonded to three other carbon atoms through *sp*^2^ hybridized orbitals. As a result, graphene holds tremendous promise for various applications, such as field-effect transistors (FETs), gas and biomolecule sensors, transparent conductive films (TCFs), and graphene batteries, due to its outstanding properties. Furthermore, when carbon atoms are arranged in layers, graphene oxide (GO) is formed, which features oxygen-containing functional groups, such as =O, -OH, -O-, and -COOH, connected to the sides and edges of the plane [49]. GO can be categorized based on its structure, which includes single-layered, bi-layered, few-layered, and multilayered structures with five to ten carbon layer arrangements. Graphite oxide refers to GO with more than ten layers of arranged carbon in two dimensions.

With the presence of multiple oxygen functional groups on its surface sites, GO can serve as a precursor for synthesizing various graphene derivative products, such as fluoro-graphene, bromo-graphene, and graphene. In addition, it is possible to synthesize graphene by thermally or chemically reducing GO. It can be noted that GO can also be applied to advanced applications, which include delivery of medicine, thermal conductivity, and materials for architecture. However, there are certain challenges with GO, such as toxicity and issues during mass production, that must be fixed and investigated in detail to utilize them for commercial applications. Thus, novel techniques for environment-friendly, inexpensive, and mass production of GOs are crucial. British chemist B. C. Brodie made the first effort to fabricate GO in 1859 while experimenting on the reactivity of flake graphite [50,51]. This method utilizes potassium chlorate as an oxidizing agent and is known as the chlorate pathway. This is pioneering work by the researcher to use a variety of potential oxidizing chemicals to break down the structure of graphite. In this method, the graphite was heated up to 60 °C for 4 days while treated with a solution of potassium chlorate and fuming nitric acid (Brodie’s graphene oxide, or BR-GO). Later, the BR-GO was subjected to several successive oxidative treatments [52,53], and the final composition of carbon, oxygen, and hydrogen was calculated to be C_11_H_4_O_5_ (corresponding to a C/O ratio of 2.2) [51].

Further, Staudenmaier and co-workers altered Brodie’s procedure (ST-GO—graphene Staudenmaier’s oxide) by altering the introduction method of the chlorate and the addition of sulfuric acid to the mixture. In this method, potassium chlorate was gradually added to the mixture to reduce the risk of explosive byproducts and the evolution of heat. Furthermore, reaction time was reduced to increase the acidity of the environment, and the resultant GO closely resembles BR-GO in terms of its characteristics. Back in 1937, Hofmann synthesized graphene oxide (HO-GO) with a reduced oxygen content (C/O ratio of 2.5) by mixing non-fuming nitric acid and potassium chlorate. Jankovský et al. (2017) reported that nitric acid levels have a substantial impact on the degree of oxidation in the graphene-based product [54].

**Figure 1:**
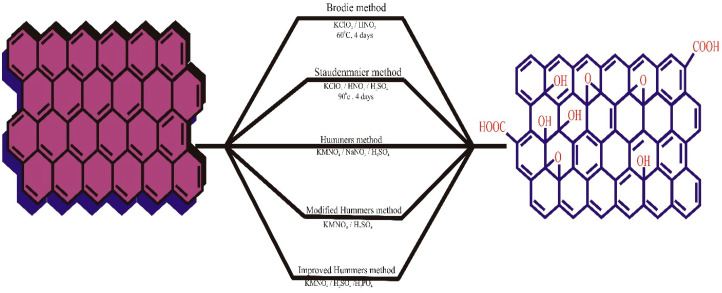
The graphical illustration of GO synthesis. Reproduced with permission from [56], ©Elsevier, 2022.

The Hummers method, also known as the permanganate procedure, is the most commonly used and efficient method for synthesizing GO to date, as developed by Hummers and Offeman in 1958. This method involves adding an excess of potassium permanganate, a low volume of sulfuric acid, and excess sodium nitrate to the reaction mixture, with a reaction time ranging from 8 to 12 h. This technique is safer than other traditional methods as it avoids the production of explosive chlorine oxide. The procedure is completed by adding a diluted solution of hydrogen peroxide (H_2_O_2_) to neutralize the excess potassium permanganate. The final product produced by the Hummers method has a C/O ratio comparable to BR-GO (2.25). However, the method is not environmentally friendly due to the presence of NOx during the reaction. Recently, Perumal et al. (2022) summarized the extracts of plants from different taxonomy groups for the novel synthesis of graphene via a green reduction approach. The authors mentioned that several plant species and major plant parts from each plant have exclusive potential to form GO and reduced GO [55]. However, these novel bio-assisted synthesis methods to form environment-friendly GO must be explored further to identify their actual mechanisms. The synthesis of GO from different methods is depicted in Figure 1.

## 4. Mechanism of Graphene Nanomaterial Formation

**Figure 2:**
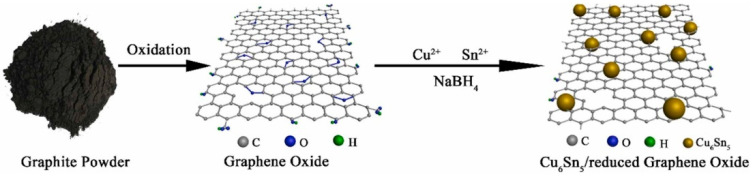
Schematic of the chemical reduction method for the fabrication of graphene oxide-bimetallic nanomaterials. Reproduced with permission from [68], ©Elsevier, 2022.

It is noteworthy that graphene oxides possess certain limitations, such as sturdiness and the inability to blend with other materials to form composites or alloys, especially for environmental applications [22]. Thus, researchers focused on the fabrication of nanosized GOs to exhibit unique properties due to their high surface-to-volume ratio compared to micro- or macro-sized GOs. In general, top-down approaches, such as liquid-phase [57], electrochemical exfoliation [58], and chemical reduction [59], and bottom-up approaches, namely chemical vapor deposition [60], epitaxial method [61], and aromatic molecule-based chemical synthesis [62], are the most common methods for the fabrication of graphene nanomaterials [63]. Gerosa et al. (2018) utilized liquid-phase exfoliation and droplet approaches for the synthesis of black phosphorus and reduced graphene oxide polymer-based saturable absorbers. In this study, multilayers of reduced-graphene oxide (r-GO) were prepared via modified Hummers’ method using N-methyl pyrrolidone (NMP). Later, black phosphorus was also exfoliated in NMP under nitrogen flow in an inert environment using an ultrasound bath, and the exfoliated samples were transformed into two-dimensional nanomaterial-based saturable absorbers via the droplet method [64]. The advantages of liquid-phase exfoliation approaches are that it is safe and easy to synthesize high-quality graphene nanomaterials with less cost, whereas the requirement of a long sonication time and low graphene concentration are their limitations [65]. Recently, Chang et al. (2022) demonstrated the fabrication of GO nanomaterials via a one-step electrochemical exfoliation method. In this study, 10 × 40 mm of carbon fiber cloth was used for the preparation of carbon (GO) nanoparticles. The cloth was immersed in an electrochemical cell with sodium hydroxide electrolytes as the working electrode, silver or silver chloride as the reference electrode, and a platinum wire as the auxiliary electrode. The resultant GO nanomaterials were formed as quantum dots with a size of ~2 nm [66]. This method leads to the formation of highly pure GO nanomaterials with high yields and comparatively large graphene sheets compared to liquid-phase exfoliation. However, the presence of impurities in the form of unwashed salt and an uncontrollable thickness parameter are the limitations of the electrochemical exfoliation approach [67]. Similarly, Lin et al. (2022) synthesized hybrid non-noble bi-metallic (Cu_6_Sn_5_) nanoparticles with reduced graphene oxide via a chemical reduction approach. The GO was synthesized via modified Hummers’ method using graphite, whereas bi-metal-GO nanocomposite was formed via chemical reduction method as shown in Figure 2 [68]. Mild conditions, easy control of the reduction process, and low chemical residuals are the advantages of the chemical reduction approach [69], whereas the usage of toxic reductants such as sodium borohydride and hydrazine, as well as the aggregation of GO in aqueous solutions, are the limitations [70].

Recently, Ullah et al. (2021) prepared size-controlled aluminum-doped graphene via a chemical vapor deposition (CVD) approach. In this study, the graphene was prepared with the help of low (23 Pa) pressure on the surface of a copper foil as a growth substrate in a horizontal tube furnace. The results showed that the CVD approach is beneficial for the preparation of high-quality aluminum-doped monolayer graphene with large areas of aluminum doped on the graphene lattice [71]. In general, the advantage of using CVD is that it produces high-quality graphene with imperviousness, homogeneity, high purity, and excellent control over layers. However, high equipment costs, toxic by-products, and high sensitivity, which are influenced by alterations in the parameters, are the limitations of CVD [72]. Further, Rodner et al. (2020) prepared graphene layers via epitaxial growth and the silicon sublimation process on the surface of silicon carbide that are decorated with several metal oxide (oxides of copper, vanadium, iron, and zirconium) nanolayers. In this method, the transfer of the graphene lattice is not required as a semi-insulating 4H-silicon carbide substrate [73]. It is noteworthy that the non-requirement of substrate transfer, the formation of high-quality GO with low defects, and seamless integration are the advantages of the epitaxial growth approach, whereas the involvement of high cost and the formation of multilayered graphene with uncontrollable size are their limitations [74]. Furthermore, Tan and Hu (2017) prepared GO via the modified Hummers’ method, which was modified with beta-cyclodextrins with the help of hydrazine under constant stirring and temperature. The study showed that the synthesis has led to the formation of a relatively smooth GO surface with few wrinkles, whereas the modified GO with cyclodextrins has a rougher surface and several wrinkles [75]. This method helps in the synthesis of unoxidized GO; however, it leads to certain contamination, which is its limitation [76]. Each synthesis approach involves a specific mechanism for the formation of GO nanomaterials. Recently, Kang et al. (2020) identified a unique formation mechanism for graphene quantum dots that are synthesized using multiwalled carbon nanotubes (MWCNTs) via pulsed laser fragmentation in liquid (PLFL). The study showed that the PLFL process has led to the structural transformation of MWCNTs, which has led to their partial destruction. Later, the quantum dots are formed near the CNT’s surface with an increase of 0.75 J of pulse laser energy. The resultant GO quantum dots were highly crystalline in nature with hexagonal graphene structures. Later, the pulsed laser energy was increased to 1 J, which led to the formation of GO quantum dots on the outer walls of MWCNTs. For a long time, the substantial laser energy has led to the disappearance of MWCNT’s wall, and only GO quantum dots exist with uniform morphology [77].

## 5. GO-Based Phenol Treatment

The removal of phenol via adsorption has received attention in the past decade. In particular, the utilization of graphene-based adsorbents for the decontamination of these organic pollutants is gaining significant attention among researchers. The treatment of phenol using graphene-based composites has been reported in various studies, as summarized and presented in Table 1.

Recently, Al-Ghouti et al. (2022) reported the preparation of GO via two methods, such as the addition of H_2_SO_4_ and HPO_4_ (named GO1) as well as H_2_SO_4_, H_3_PO_4_, and HNO_3_ (named GO2) for the treatment of phenol in water. The influence of various parameters, including initial phenol concentration, temperature, and pH, on the adsorption process was also studied. The authors reported the modeling of the equilibrium adsorption data and compared it with conventional models, such as Freundlich, Langmuir, Temkin, and Dubinin-Radushkevich adsorption isotherm models. It was noted that ultraviolet (UV) ray irradiation had a favorable influence on the adsorption process while increasing their adsorption capacity. It can be noted that the maximum adsorption capacities for GO1 and GO2 were 0.77 and 20.2 mg/g, respectively. It has been reported that the adsorption capacity reached its peak at pH 2 in the absence of UV radiation, whereas pH 6 was identified as optimal in the presence of UV irradiation. The adsorption rate increased with an increment in the initial phenol concentration, and an equilibrium point was reached where there was a drop in adsorption, after which it remained constant. The phenol concentration increment has resulted in increased pollutant uptake until equilibrium is attained, where the adsorption is reduced and has maintained a constant value. The maximum adsorptive capability of GO1 and GO2 has been elevated from 70.43 to 90.82% and 86.75 to 95.95% after UV exposure, respectively [9]. Further, Parvin et al. (2022) studied the fabrication of graphene oxide-magnetic/polyrhodanine (GO-Fe_3_O_4_/PRd) for the adsorption of phenol in a batch process from a hydrous solution. The maximum adsorptive capability of GO composite was 191.0 mg/g at 15 min of equilibrium time. The adsorption behavior of phenol by the GO-Fe_3_O_4_/PRd adsorbent was described by Freundlich and Langmuir isotherm models with a pseudo-second-order kinetic model. The authors also revealed that the uptake of phenol by GO-Fe_3_O_4_/PRd was spontaneous, endothermic, and feasible [78].

Bibi et al. (2022) developed GO modified with polyacrylic acid (GO-PAA) as a composite for the removal of phenol from real and synthesized effluents. The author utilized a variety of techniques, such as scanning electron microscope (SEM) and energy dispersive X-ray analysis (EDX), as well as Fourier transform-infrared (FTIR) and Brunauer-Emmet-Teller reaction (BET) techniques, to examine the GO and GO-PAA samples’ morphology, surface area, and surface chemistry, respectively. The adsorption parameters, such as temperature, pH, and initial phenol content, were also evaluated. The best adsorption was identified at a pH of 2 and a temperature of 25 °C. The results showed that the modification of the GO-PAA surface can lead to the aggregation of C=O groups, which eventually increases the adsorption ability of unmodified GO. The study also revealed that Langmuir’s isotherm model was best suited to describe the adsorption process of the GO sample, and the thermodynamics studies proved the exothermic and spontaneous nature of phenol adsorption processes. It was observed that the GO-PAA sample was able to remove 75% of the phenol from synthetic wastewater and 18% from real wastewater, respectively, at an optimal pH of 2 and a temperature of 25 °C [26]. Further, Zhao et al. (2021) identified that conjugated graphene-based materials can be utilized to mitigate wastewater that has been polluted by the presence of phenolic compounds. The study revealed the adsorption mechanism and optimized the treatment efficacy of p-nitrophenol as well as phenols by developing a nitrogen-doped reduced graphene oxide (N-RGO) with a broadened aggregated π region that is enlarged via annealing a composite that is made up of chitosan decorated in a GO matrix via an exfoliation process. The results indicated that the maximum phenol and p-nitrophenol uptake by N-RGO was 155.82 and 80.60 mg/g, respectively, at an optimal 200 mg/L initial concentration, pH of 6, and 30 °C temperature. The π-π and hydrophobic interactions were identified as being responsible for the improved removal efficiency. Furthermore, N-RGO was observed to retain a high adsorption capacity (>80%) from regeneration experiments over five cycles. These results emphasized that N-RGO is an excellent material as an effective and regeneratable sorbent for the adsorption of phenolic compounds [27].

Zhou et al. (2020) synthesized a three-dimensional amino-poly(vinylamine) (PVAm)-functionalized GO-(o-MWCNTs)-Fe_3_O_4_ composite via a one-pot process for phenol uptake in batch adsorption. The composite exhibited a huge surface area and copious amino groups, and the maximum adsorptive capability, evaluated from the Langmuir model under optimum conditions, was 224.21 mg/g, with equilibrium reached in 60 min. The adsorption mechanism involved π-π interactions, hydrogen bonding, and electrostatic interactions between PVAm and phenols, and the composite was loaded with phenols that could be easily recycled using NaOH solution. Thus, the PVAm-GO-(o-MWCNTs)-Fe_3_O_4_ composite was an effective adsorbent for phenolic compound removal [79]. In another study, Badhai et al. (2020) developed a GO-Fe_3_O_4_ hybrid material using sonication-assisted co-precipitation techniques to treat phenol. The adsorption kinetics showed that the phenol uptake efficacy was 68% in aqueous systems, with an outstanding adsorptive capability of ∼79 mg g^−1^ and an initial adsorption rate of 5.7 mg g^−1^ min^−1^. The process was initially governed by the boundary, then by surface diffusion, and finally via pore diffusion, as indicated by the kinetic models. According to the Langmuir isotherm, equilibrium isotherms showed that the adsorption was a spontaneous physisorption process with a maximum adsorption capacity of 658 mg/g [80]. Manna et al. (2019) treated phenol from wastewater using GO particles coated with biochar. The maximum adsorptive capability of phenol was 23.47 mg, and the equilibrium data were best explained using the Langmuir isotherm via phenol disintegration in monolayer surface adsorption. The kinetic model studies showed that the process of phenol disintegration was proceeded by pseudo-second-order kinetics, and diffusion played a crucial role in the adsorption process, as evident from the intra-particle diffusion model [81]. All these studies indicate that GOs (micro and macro-sized) possess enhanced ability for phenol adsorption in wastewater, but the adsorption efficiency can be improved by blending GOs with other materials.

**Table 1:** Summary of phenol adsorption onto various graphene-based composites.

Adsorbents	Experimental Conditions			Adsorption Capacity (mg/g)	Isotherm Models	Kinetics Models	Adsorption Mechanisms	References
	pH	Time (min)	Temp. (°C)					
GO1	2	-	35	0.9	Langmuir	-	Hydrophobic effect, electrostatic interaction, H-bonding, π-π-interaction, and van der Waals forces	[9]
GO2	6	-	25	20.2				
GO-Fe_3_O_4_/PRD	7	15	40	191	Langmuir	PSO	-	[78]
GO-PAA	2	-	25	84	Langmuir	-	π-π interaction, electrostatic, and hydrophobic interaction, H-bonding, dispersion by van der Waals forces	[26]
GO-coated biochar	7	60	35	23.47	Langmuir	PSO	-	[81]
GO-PNIPAM	7	-	25	12.74	Langmuir	-	H-bonding	[82]
GO-(O-MWCNTs)-Fe_3_O_4_	6	60	-	224.21	Langmuir	PSO	H-bonding, electrostatic interaction, hydrophobic, and π-π interaction	[79]
GO/PPy	5	1440	-	7.75	Langmuir	PSO	Ion exchange, π-π -electron donor acceptor (EDA) interaction, hydrophobic interaction, and Lewis’s acid-base interaction	[83]
-N-RGO	6	2160	30	155.82	-	PSO	Electrostatic, hydrophobic, and π-π interactions	[27]
GO-Fe_3_O_4_	4	70	-	657.9	Langmuir	PSO	-	[80]
GO	7	-	30	10.23	Langmuir	PSO	-	[84]
GO-CTES-β-CD/PNIPAM	7	-	25	131.64	Freundlich	PSO	Hydrogen bonding	[24]

## 6. Novel Graphene Nanomaterial-Based Concepts for Phenol Treatment

The limitations of micro- and macro-sized graphene materials have led to the emergence of graphene nanomaterials and nanocomposites for the treatment of phenols via adsorption. Maio et al. (2020) prepared three-dimensional templates with graphene oxide-decorated polycaprolactone (PCL) via a one-pot process. In this study, the PCL solutions were coated on GO nanoparticles in ethanol through jet-wet electrospray or electrospinning on the surface of a stirred liquid collector. The phenol removal analysis showed that the GO-PCL nanoparticles possess a sorption equilibrium of 6 h via a pseudo-second-order model in fresh water with excellent robustness and recyclability [85]. Even though the resultant GO nanoparticle helped in the adsorption of phenols from the wastewater, the use of hazardous reducing agents to form GO has the ability to cause toxic reactions in the environment. Thus, Haydari et al. (2023) recently fabricated reduced GO using *Verbena officinalis* to reduce their toxicity towards the environment. The resultant GOs were encapsulated in sodium alginate via cross-linking to utilize the system for phenol treatment in olive mill wastewater. The study showed that these green synthesized GOs in sodium alginate were identified to possess 3.68 g/L of adsorbent dosage at a temperature of 25 °C and a pH of 4 with 135 min of adsorption time. In addition, the study also revealed that the GO system possesses 994 mg/g of phenol adsorption capacity for 4000 mg/L of initial concentration [48]. However, these green synthesized GO nanomaterial systems lack stability for a long time, which necessitates the requirement for a GO composite system for phenol treatment in the actual environmental conditions.

**Figure 3:**
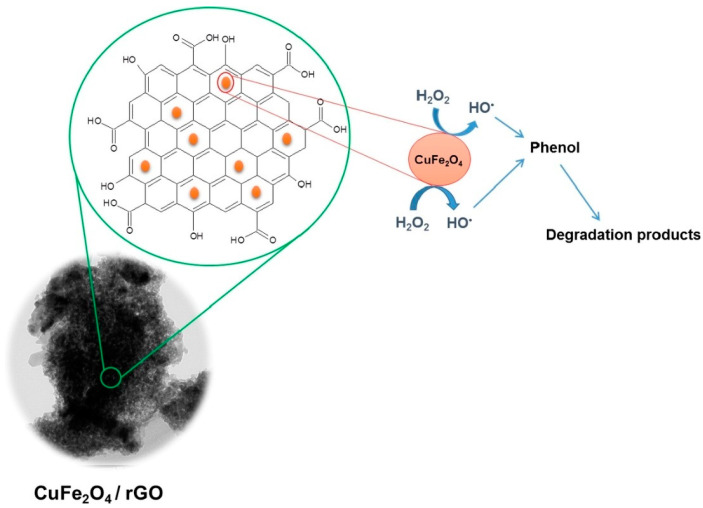
Schematic mechanism of graphene nanocomposite for the effective degradation of phenol in wastewater. Reproduced with permission from [86], ©Elsevier, 2019.

Othman et al. (2019) prepared novel copper-iron oxide nanoparticles that are supported on the surface of the reduced GO via a facile low-temperature approach. The synthesis has led to the decoration of copper-iron oxide nanoparticles on the surface of reduced GO sheets. The study showed that the composite possesses enhanced photocatalytic activity for phenol degradation. The results showed that the GO system can completely remove phenol from the contaminated water within 15 min at room temperature under photocatalytic conditions, as shown in Figure 3 [86]. Further, Kumar et al. (2021) fabricated a novel nanocomposite with reduced GO and zinc oxide tetrapods via refluxing. The study identified that the reduced GO nanosheet is responsible for enhancing the production of reactive oxygen species by zinc oxide tetrapods. The results emphasized that the nanocomposite possesses an enhanced ability to photodegrade 94.8% of 4-chlorophenol under ultraviolet (UV) ray exposure within 180 min. Similarly, 98.05% of methylene blue dye was identified as being photodegraded by the nanocomposite after UV light irradiation within 90 min [87]. Recently, Wu et al. (2023) prepared a novel membrane with iron nanoparticle-doped reduced GO for the effective removal of micropollutants from water. The study showed that the resultant membrane possesses a water permeance level of 39.8 L/m^2^/h/bar, which is higher than individual graphene oxide membranes. The results revealed that the membrane can remove >75.8% of p-nitrophenol during a dead-end ultrafiltration process from wastewater [88]. All these studies showed that the GO-based nanocomposites possess an enhanced ability to remove phenolic pollutants from wastewater compared to standalone GO nanoparticles. However, there are only a few studies that demonstrate the efficiency of GO nanoparticles for phenol removal from POME. Recently, Lawal et al. (2020) utilized a one-step steam pyrolysis process for mesoporous biochar production from oil palm fronds, which contain graphene, for effective phenol removal in facultatively treated POME. The biochar with graphene possesses an enhanced ability to remove 90% of phenol from a 16–20 g/L dosage of treated POME with zero percentage of aerobic microbial growth inhibition [2]. Thus, more studies should be directed in this direction, especially with GO-based nanocomposites to improve phenol contaminant removal from POME-containing wastewater.

## 7. Future Perspective

The development of efficient, environmentally friendly, and cost-effective GO-based nanomaterials for micropollutant adsorption has gained considerable interest among researchers, industrial sectors, and policymakers [89]. However, further research is required to enhance the phenol removal efficiency of GO nanomaterials for commercial applications, including the characterization and evaluation of the adsorption behavior of various micropollutants with different partition coefficients [90,91]. Novel synthesis methods, such as biological methods using plant or microbe-extracted GO nanomaterials or hybrid approaches linking biological and chemical methods [92], as an alternative to conventional synthesis approaches, can be used to fabricate graphene-based materials to increase their selectivity and adsorption of specific micropollutants without leading to any toxic reactions in the environment [93]. Various criteria, such as raw material availability and cost, surface area, porosity, volume of pores, surface chemistry, and regenerability, are considered in the fabrication of adsorbents [94]. However, some of these criteria are not adequately discussed in most studies, leading to critical research gaps in the commercial application of these modified GO-based adsorbents. Thus, foundational research can be leveraged to produce design models for future studies, predict their practical effectiveness, optimize their design, and provide economic evaluations of the adsorption process. Desorption and regeneration are crucial processes to consider from an economic standpoint when using graphene-based adsorbents [95]. These adsorbents must be separated from the adsorbate and regenerated for reuse [96]. Further, these GO nanomaterials can be combined with carbon nanofibers to be beneficial for the purification of water that contains phenol [97]. Moreover, practicality and environmental sustainability are essential for GO-based adsorbents to be considered a cost-effective option in the future [98]. In general, degradable GO nanomaterials must be used for phenol removal to avoid the possible toxicity of nanomaterials to the environment. In addition, magnetic nanomaterials can be combined with GO as nanocomposite, with the ability to collect them via magnets after the removal of phenol. However, these concepts of environmentally safe nanomaterials are still under extensive investigation and can be novel nanomaterials for environmental remediation applications in the future. It should be noted that all the studies reviewed were conducted at a laboratory-scale level. Therefore, further studies should focus on the use of these adsorbents for wastewater decontamination at large-scale and contaminated sites. An ideal adsorbent should possess high adsorptive capability and excellent desorption properties, making it highly cost-effective and proficient [99]. Therefore, GO-based nanocomposites have the potential to be developed as effective phenol adsorbents for wastewater in the future.

## 8. Conclusions

In conclusion, the studies conducted on GO-based nanocomposites as adsorbents for phenol removal applications have demonstrated their high efficiency and effectiveness. These nanocomposites offer several advantages, including a high surface area, excellent mechanical strength, and unique chemical and physical properties, which contribute to their superior adsorption capacity for phenol and other organic contaminants. Furthermore, kinetic studies have shown that GO-based nanocomposites typically exhibit a pseudo-second-order reaction for phenol adsorption processes, indicating their ability to rapidly and efficiently remove phenol from wastewater. Moreover, the capacity of various GO-based composites with different modification types for adsorbing phenols has been extensively reported, showcasing their versatility and applicability in diverse wastewater treatment applications. It is noteworthy that the recovery of nanomaterials is the most significant aspect of their application in environmental applications. Recent research has highlighted the importance of developing efficient methods for the recovery and reusability of nanocomposites. Although specific percentages for nanomaterial recovery depend on the process, it is a critical area of focus for future investigations. Incorporating sustainable and environmentally friendly approaches to recover and recycle these materials will enhance the overall sustainability and cost-effectiveness of phenol removal processes. In summary, GO-based nanocomposites offer a sustainable and effective solution for the treatment of phenol-contaminated wastewater, providing a cost-effective and eco-friendly approach for pollutant removal. Further research efforts should aim to develop novel and efficient materials while also focusing on the recovery and reusability of nanomaterials to optimize their overall environmental impact and resource utilization.

## Data Availability

No data were created for this study.
